# Changes in EEG Microstate Dynamics and Cognition Post‐Chemotherapy in People With Breast Cancer

**DOI:** 10.1002/brb3.70335

**Published:** 2025-03-04

**Authors:** S. Damji, S. Sattari, K. Zadravec, K. L. Campbell, J. Brunet, N. Virji‐Babul

**Affiliations:** ^1^ Graduate Program in Neuroscience, Faculty of Medicine University of British Columbia Vancouver Canada; ^2^ Graduate Program in Biomedical Engineering, Faculty of Medicine University of British Columbia Vancouver Canada; ^3^ Graduate Program in Rehabilitation Sciences, Faculty of Medicine University of British Columbia Vancouver Canada; ^4^ Department of Physical Therapy, Faculty of Medicine University of British Columbia Vancouver Canada; ^5^ School of Human Kinetics, Faculty of Health Sciences University of Ottawa Ottawa Canada; ^6^ Djavad Mowafaghian Centre for Brain Health University of British Columbia Vancouver Canada

**Keywords:** brain fog, breast cancer, cancer, chemotherapy, cognition, electroencephalography (EEG), EEG microstate analysis

## Abstract

**Objective:**

Chemotherapy‐related cognitive changes following breast cancer are commonly reported; however, changes in brain dynamics of large‐scale neural networks remain unclear. Using data from the *Aerobic exercise and CogniTIVe functioning in women with breAsT cancEr (ACTIVATE)* trial, we conducted exploratory analyses to compare self‐reported and objective measures of cognition and applied microstate analysis to resting state (RS) electroencephalography (EEG) data of women with breast cancer before and following chemotherapy treatment.

**Methods:**

Data from eight female participants between the ages of 30 and 52 (mean age = 44.8 years, SD = 7.3 years) were analyzed. Cognitive function was assessed using the PROMIS (Patient‐Reported Outcomes Measurement Information System) and the Trail Making Test (TMT). Five minutes of RS eyes‐closed EEG data were also collected. Seven EEG microstates were extracted, and mean microstate duration and occurrence were computed.

**Results:**

Following chemotherapy, there was a significant decrease in the PROMIS score (*p *= 0.003, *d *= 1.601), but no significant difference in the TMT score. Overall, microstate durations were significantly longer (*p *< 0.001, *d *= 2.837) and less evenly distributed following chemotherapy. The mean duration of microstate D (involved in attention/executive functions) significantly increased following chemotherapy (*p *= 0.007, *d* = 1.339). Comparing behavioral and microstate measures that exhibited a large effect size, no significant correlations were observed either before or after chemotherapy.

**Conclusions:**

We observed self‐reported cognitive impairment and disturbed functional dynamics in the RS brain following chemotherapy. This exploratory study provides new evidence using a within‐cohort design showing that changes occur in large scale brain dynamics related to the cognitive effects of chemotherapy.

**Trial Registration:**

ClinicalTrials.gov identifier: NCT03277898

## Graphical abstract

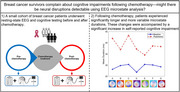

## 1 Introduction

Chemotherapy‐related cognitive impairment (CRCI), colloquially referred to as “chemo brain” or “chemo fog,” is a cluster of cognitive impairments including mental fatigue, attentional problems, and memory complaints (Anderson‐Hanley et al. 2003; Campbell et al. 2020; Kam, Brenner, et al. 2016; Squillace et al. 2022; Kim et al. 2023). Breast cancer survivors who have completed chemotherapy report greater cognitive concerns posttreatment relative to pretreatment, as well as more executive function deficits and impaired memory compared with matched healthy controls (Arya et al. 2021; Henneghan and Kesler 2023). Few studies of the neurophysiological correlates of CRCI have been conducted, and currently there is no consensus on a “brain signature” of CRCI, making it difficult to develop strategies to manage this issue (Henneghan and Kesler 2023).

Previous electroencephalography (EEG) research to study CRCI in breast cancer survivors has primarily focused on isolated EEG features, such as event‐related potentials (ERP) and static measurements of EEG band power underlying elevated physical and mental fatigue and changes in attentional processes (Kam, Brenner, et al. 2016; Melara et al. 2023; Moore et al. 2014). However, these measures do not capture changes in the functional organization of brain networks.

Probing the brain during the “resting state” (RS) (i.e., when the brain is not involved in active cognitive tasks) is a powerful method to map the functional organization of the brain. EEG studies show that RS is characterized by spontaneous, yet coherent fluctuations in electromagnetic fields from functionally distinct brain regions, and subsets of these regions tend to act in concert, giving rise to functionally relevant RS brain networks (da Cruz et al. 2020; Xu et al. 2020). These networks play a critical role in mediating complex functions such as memory, language, and emotional states (Seitzman et al. 2019; Canu et al. 2022). More recently, EEG microstate analysis has been applied to study the brain's RS by describing the discrete functional cortex‐wide states at rest. At rest, the brain does not exist in one state but shifts dynamically between four and seven different EEG “microstates” that are stable for about 20–120 ms before shifting to another state (Lehmann 1990; Lehmann, Pascual‐Marqui, and Michel 2009; Katayama et al. 2007). EEG microstates capture subtle temporal dynamics in functional brain areas and networks that cannot be captured with RS fMRI, given the limited temporal resolution of the BOLD signal. Thus, EEG is a promising approach for understanding altered dynamics of brain activity (Michel and Koenig 2018).

In EEG microstate analysis, a modified *k*‐means clustering algorithm is typically applied to segregate the multichannel time series data into fleeting epochs of cortex‐wide electrical activity patterns (i.e., EEG microstates). Although four canonical microstate templates (A–D) have been most prevalent in EEG microstate literature to date, Custo et al. (2017) argued that seven distinct microstate templates best capture the scope of spontaneous electrophysiological activity topographies observed in RS EEG studies. Microstate A is associated with the auditory network, B with the visual network, C with the salience network, D with the attention network, E with the default mode network (DMN), F with cognitive and emotional processing related to activity in the anterior cingulate cortex (ACC), and G with the sensorimotor network (Custo et al. 2017). These microstates (labeled A–G) have been reliably documented in the neuroscience literature and reproduced across numerous EEG studies (Kleinert et al. 2023).

Several studies have investigated EEG microstates to characterize neural changes that occur in specific clinical/neurological conditions, including mood and anxiety disorders (Al Zoubi et al. 2019), with mixed results (da Cruz et al. 2020; Metzger et al. 2024). In women with breast cancer, microstate features were predictive of chronic postoperative pain perception among those who underwent surgery (Li et al. 2023). To our knowledge, EEG microstate analysis has not yet been applied to understand CRCI in women with breast cancer.

An objective method to diagnose and monitor the severity of CRCI during and after chemotherapy for breast cancer would therefore be beneficial. Such a method would provide mechanistic insights, enhance understanding of women's CRCI symptoms, and inform targeted interventions to mitigate the impact of chemotherapy on specific functional areas of the brain, as well as cognitive function (Lange et al. 2019; Duivon et al. 2023; Baghdadli et al. 2023). The goal of this exploratory study is twofold: explore a new method of analyzing neural changes that occur in women receiving chemotherapy for breast cancer, and to generate specific hypotheses for future confirmatory and better‐powered investigations.

## 2 Methods

### 2.1 Participants

We analyzed data collected during 2018–2020 as part of a longitudinal randomized controlled trial entitled *Aerobic exercise and CogniTIVe functioning in women with breAsT cancEr (ACTIVATE)* (Brunet et al. 2024), which tested the effects of aerobic exercise on cognitive function and quality of life in women with Stages I–III breast cancer receiving chemotherapy. For this exploratory analysis, all participants were collapsed into a single group. Ethics approval was granted by research ethics boards at the University of Ottawa (Ottawa, ON) and the University of British Columbia (UBC) (Vancouver, BC), as well as relevant hospital research ethics committees. The trial was registered at ClinicalTrials.gov (NCT03277898; September 11, 2017). All participants received written and oral information prior to participation and provided informed consent.

### 2.2 Self‐Reported and Objective Measures of Cognitive Function

The ACTIVATE trial included several outcome measures as described in Brunet et al. (2020, 2024). Herein, we selected one self‐report measure and one objective measure pre‐ and post‐chemotherapy to capture symptoms of cognitive impairment. For the self‐report measure, we used the 4‐item PROMIS (Patient‐Reported Outcomes Measurement Information System) Applied Cognitive Abilities Scale (Saffer et al. 2015). The PROMIS Scale assesses self‐perceived cognitive function over the past 7 days regarding areas of concentration, mental acuity, and memory. It consists of four positively‐worded items: (1) “Has your mind been as sharp as usual?” (2) “Has your memory been as good as usual?” (3) “Has your thinking been as fast as usual?” and (4) “Have you been able to keep track of what you are doing, even if you are interrupted?” Response choices range from “not at all” (1) to “very much” (5), coded to a 1–5 Likert Scale with scores ranging from 1 to 20.

For the objective cognitive function measure, we used the Trail Making Test (TMT), a standard assessment of cognitive flexibility (i.e., the ability to switch attentional resources between different tasks) (Reitan 1955). The TMT consists of two separate scores for the time (in seconds) taken to complete two visual search tasks (i.e., part A and part B) that require alternating attention in the most efficient manner possible. Higher PROMIS scores reflect a stronger subjective appraisal of cognitive function, whereas higher TMT scores (i.e., longer times) correspond to poorer cognitive flexibility.

### 2.3 EEG Data Collection and Preprocessing

EEG data collection was optional for participants and only offered at one study site (UBC). Baseline EEG testing was performed prior to chemotherapy (mean = 4.3 ± 6.1 days before chemotherapy). Post‐chemotherapy (i.e., after the completion of the last chemotherapy treatment) EEG testing was a mean of 25.3 ± 18.4 days after chemotherapy. All data was collected at the Perception‐Action laboratory at UBC. Participants were asked to sit comfortably in a chair with their eyes closed and instructed not to move, to relax and not think of anything in particular. Five minutes of RS EEG data were collected. A 64‐channel EGI HydroCel Geodesic SensorNets (EGI, Eugene, OR) using the Net Amps 300 with 500 Hz sampling rate and Cz as the reference was used. Scalp electrode impedances were kept below 50 kΩ.

Raw EEG data were preprocessed using EEGLAB (v2023.1) (Delorme and Makeig 2004) in MATLAB (v2023b). Each participant's EEG was re‐referenced to the average of all channels. A notch filter at 60 Hz, a low‐pass filter at 50 Hz, and a high‐pass filter at 0.5 Hz were applied to the EEG time series. Independent Component Analysis (ICA) was used to identify and remove any non‐brain artifacts identified through a combination of visual inspection and EEGLAB's ICLabel classification function (Pion‐Tonachini, Kreutz‐Delgado, and Makeig 2019).

### 2.4 EEG Microstate Analysis: Mean Duration and Mean Occurrence Frequency

EEG microstate analysis was performed in MATLAB (v2023b) using the MICROSTATELAB toolbox (v1.0) (Nagabhushan Kalburgi et al. 2023). Mean microstate maps were first generated for each participant at both timepoints, mapped onto the widely used microstate templates, and then backfitted (i.e., the raw EEG was re‐expressed as a sequence of microstate classes) to each participant's EEG time series for feature extraction. As per Nagabhushan Kalburgi et al. (2023), grand mean maps were used as the template for backfitting to ensure optimal comparability across participants and the most conservative analysis of extracted microstate features. As there is no consensus in the literature on determining the optimal number of classes to use for EEG microstate analysis (da Cruz et al. 2020), we sought a cluster solution that minimized globally explained variance (GEV) and extracted seven microstate classes.

**FIGURE 1:**
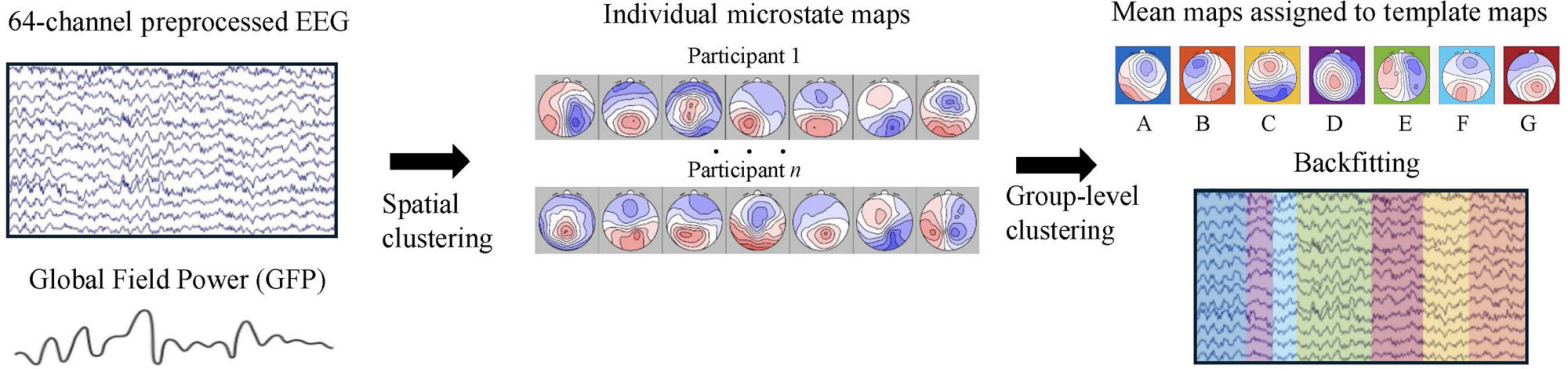
Flowchart outlining the microstate analysis process. Global Field Power (GFP) is first computed from an individual participant's preprocessed EEG data. Next, electric potential topographical maps are derived at the GFP peaks. Individual‐level spatial clustering using the *k*‐means algorithm is performed on these topographical maps, yielding individual microstate maps. Following this, a second clustering process is applied across all individuals to generate group mean microstate maps. Mean maps are then sorted to normative template maps (A–G) based on shared variance. Finally, backfitting of these template maps at the subject level occurs, enabling the extraction of temporal features associated with individual microstates.

Backfitting was performed using seven microstate classes on Global Field Power (GFP) peaks of the EEG recording. Microstate labels were interpolated in between these maxima using the nearest neighbor criterion, based on the most closely associated GFP label (Custo et al. 2017). The mean duration (reflecting the temporal stability of a particular microstate) and the mean frequency of occurrence per second (the tendency of a particular microstate to be active) were extracted for each microstate. Figure 1 summarizes this process.

### 2.5 Statistical Analysis

*PROMIS and TMT data*: Group‐level paired *t*‐tests were performed to detect changes before and following chemotherapy. A Bonferroni‐adjusted significance level of 0.017 was calculated to account for the increased probability of type‐I error.

*EEG data*: For each timepoint, we computed the mean duration and occurrence frequency for each of the seven analyzed microstates. A paired sample *t*‐test was first performed on the average duration of all microstates. We then conducted seven *t*‐tests for microstate duration and seven for occurrence frequency and adjusted the significance level for each test to 0.007 to account for multiple comparisons.

Pearson correlation tests between microstate features and measures of cognitive function that showed a large effect size (*d *≥ 0.8) between timepoints were performed to assess possible linear brain–behavior linkages in the data. The goal of this exploratory correlation analysis was to assess whether the most notable changes observed in brain activity topography could be mapped onto the most notable indicators of cognitive impairment observed before versus after chemotherapy.

## 3 Results

Of the 37 individuals enrolled at the UBC site, 10 female participants completed the optional RS EEG scan along with the required battery of behavioral and neuropsychological tests before (i.e., baseline) and after (i.e., post‐chemotherapy) chemotherapy. One participant was removed as their EEG data was too noisy for adequate preprocessing and analysis; an additional participant was not able to complete post‐chemotherapy EEG testing due to COVID restrictions. Eight participants (mean age = 44.8 years, SD = 7.3 years) were included in the final analysis for this study. One participant had their baseline EEG scan performed 1 week after starting chemotherapy, prior to completing their first 3‐week chemotherapy cycle. Four participants were in the exercise group and four in the delayed exercise group. Table 1 summarizes the sociodemographic and clinical characteristics of the analytical sample.

**TABLE 1:** Demographic, clinical, and scan timing characteristics of the cohort (*n* = 8).

Demographic/clinical variable	Group summary
Age in years (mean ± SD)	44.8 ± 7.3
Ethnicity (Caucasian/Chinese)	5/3
Breast cancer stage (1/2/3)	3/3/2
Chemotherapy type^a^ (ACT/ACT‐G/DC)	4/1/2
Weeks of chemotherapy (mean ± SD)	16.0 **± **3.7
Days between baseline scan and chemotherapy start (mean ± SD)	4.3 **± **6.1
Days between post‐chemotherapy scan and chemotherapy end (mean ± SD)	25.3 **± **18.4

^a^ One participant's chemotherapy type could not be retrieved.

Abbreviations: ACT = doxorubicin, cyclophosphamide, paclitaxel; ACT‐G = agents in ACT + filgrastim; DC = docetaxel, cyclophosphamide.

### 3.1 Self‐Reported and Objective Measures of Cognitive Function

Pre‐ and post‐chemotherapy scores are reported in Table 2. At the Bonferroni‐adjusted significance level, the PROMIS score significantly decreased following chemotherapy and demonstrated a large effect size (*p *= 0.003, *d *= 1.601). Overall, participants self‐reported significantly impaired memory, cognitive speed, and ability to focus on tasks following chemotherapy.

**TABLE 2:** Neurobehavioral outcome data at baseline and following chemotherapy treatment (*n* = 8).

Behavioral/EEG outcome variable	▲/▼	Mean ± SD (baseline/post‐chemotherapy)	*p* value	Effect size	95% CI
^**^PROMIS score	▼	14.3 ± 5.0/10.6 ± 4.6	*p* = 0.003	*d* = 1.601	[−5.52, −1.73]
TMT part A score	▼	31.5 ± 10.5/26.9 ± 6.9 s	*p* = 0.283	*d* = 0.411	[−13.97, 4.76] s
TMT part B score	▼	67.2 ± 22.3/55.6 ± 12.0 s	*p* = 0.158	*d* = 0.558	[−29.01, 5.78] s
Microstate A duration	▲	28.2 ± 5.7/42.0 ± 9.1 ms	*p = *0.016	*d = *1.120	[3.51, 24.15] ms
Microstate C duration	▲	30.3 ± 6.1/43.3 12.0 ms	*p = *0.044	*d = *0.869	[0.50, 25.47] ms
^**^Microstate D duration	▲	26.9 ± 7.4/37.2 7.0 ms	*p* = 0.007	*d* = 1.339	[3.87, 16.75] ms
Microstate G duration	▲	29.3 ± 8.3/38.3 ± 7.3 ms	*p = *0.043	*d = *0.875	[0.40, 17.67] ms
Microstate B occurrence	▼	5.1 ± 2.1/2.9 0.7 Hz	*p = *0.018	*d = *1.090	[−3.82, −0.50] Hz
Microstate F occurrence	▼	5.1 ± 1.6/3.6 ± 1.2 Hz	*p = *0.029	*d = *0.971	[−2.66, −0.20] Hz

*Note*: Cognitive function test results and notable paired‐sample *t*‐test results comparing microstate duration and occurrence frequency at baseline and post‐chemotherapy are reported.

** statistically significant result given the appropriate Bonferroni‐adjusted significance level.

Neither of the TMT scores following chemotherapy were significantly different from scores before treatment (part A: *p *= 0.283, *d *= 0.411; part B: *p *= 0.158, *d *= 0.558). Both scores decreased (i.e., corresponding to shorter times and thus better performance on the TMT) following chemotherapy, with a small effect size observed for the time to complete part A and a medium effect size observed for the time to complete part B. These effect sizes indicate participants’ performance on the TMT slightly improved following chemotherapy.

### 3.2 EEG Microstate Results

**FIGURE 2:**
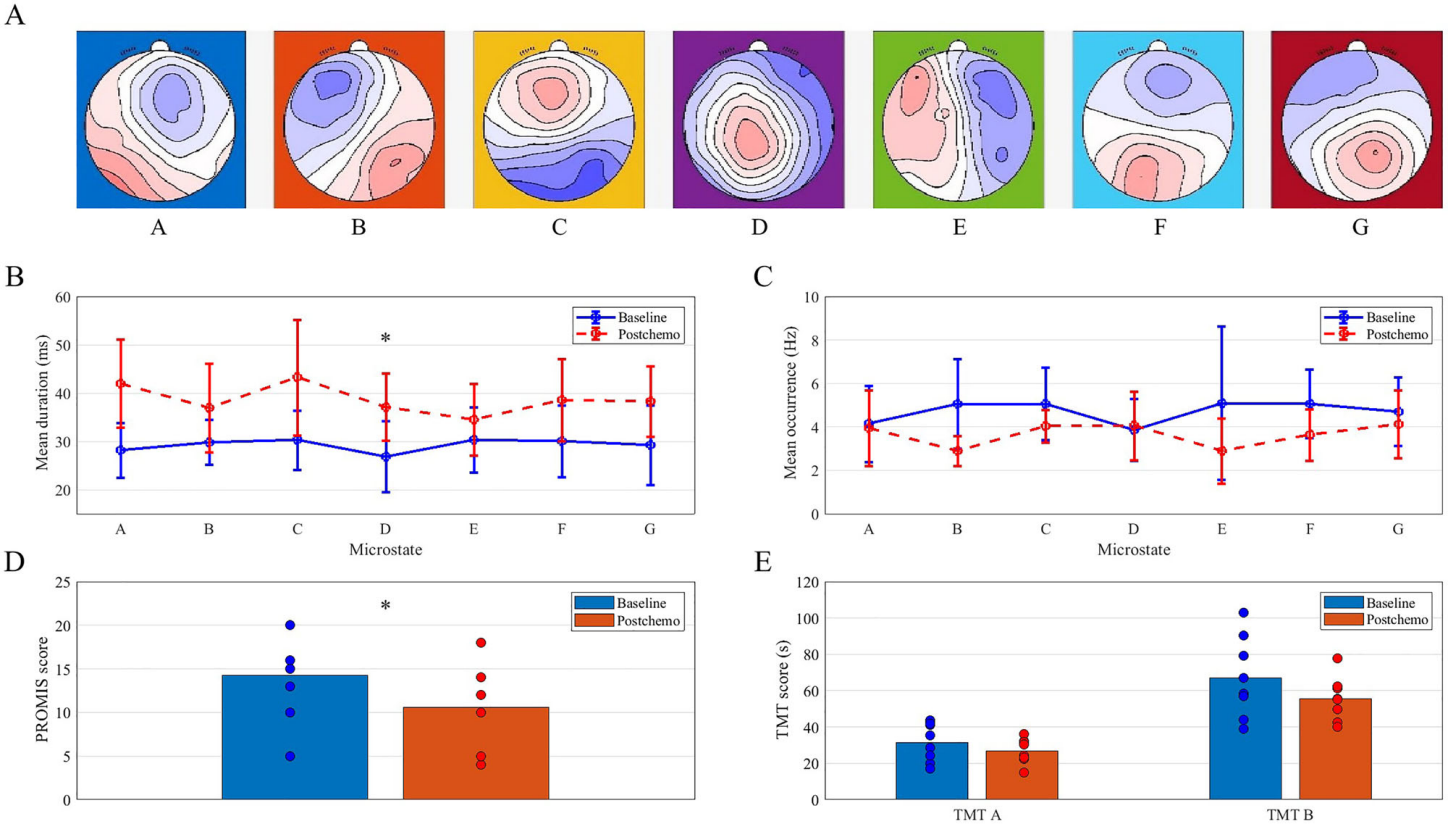
Overview of key results. (A) Mean microstate maps generated for our cohort and sorted to the normative A–G maps. (B) Overall, mean microstate durations following chemotherapy were significantly longer (*p *< 0.001, *d *= 2.837) and more than two times more variable (SD = 3.02) than mean microstate durations in the baseline group (SD = 1.31). The mean duration of microstate D was significantly longer following chemotherapy (*p = *0.007*, d = *1.339). (C) There were no statistically significant results regarding the mean occurrence frequency of the microstates. (D) The PROMIS Cognitive Abilities total score was significantly reduced following chemotherapy treatment (*p *= 0.003, *d *= 1.601). (E) No statistically significant differences were observed in TMT scores between baseline and post‐chemotherapy.

**FIGURE 3:**
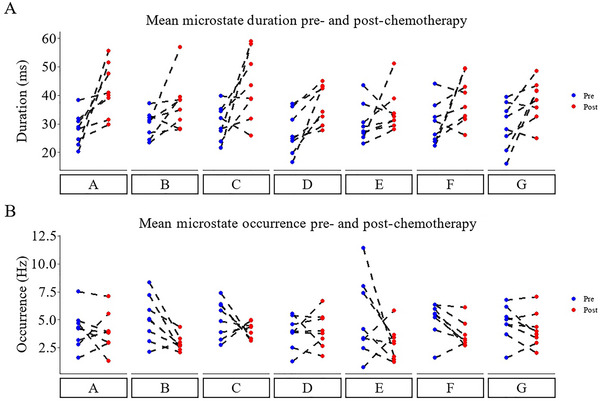
Individual subject‐level changes in mean microstate duration (A) and occurrence (B) before and after chemotherapy treatment. Blue dots represent pre‐treatment data, while red dots represent posttreatment data, with dashed lines connecting each subject's pre‐ and posttreatment measurements.

Figure 2 visually depicts the group‐level results for individual microstate mean duration and mean occurrence pre‐ and post‐chemotherapy. Figure 2A shows the mean microstate maps generated for the cohort, organized based on the normative A to G maps. Figure 3 depicts individual subject‐level changes.

### 3.3 Microstate Mean Duration

Overall, the mean duration of all microstates post‐chemotherapy significantly increased (Figure 2B) and exhibited a large effect size (*p *< 0.001, *d *= 2.837). The range of mean durations for the seven microstates was narrower at baseline (range: 26.9–30.3 ms) compared to post‐chemotherapy (range: 34.5–43.3 ms). The variability in mean microstate durations was more than twice as high post‐chemotherapy (SD = 3.02) compared to pre‐chemotherapy (SD = 1.31).

Only the mean duration of microstate D showed a statistically significant increase following chemotherapy (*p = *0.007*, d = *1.339) (Figure 2C). Based on the observed large effect size, however, other results of practical significance are the increase in the mean duration of microstate A (*p = *0.016*, d = *1.120), microstate G (*p = *0.043*, d = *0.875), and microstate C (*p = *0.044*, d = *0.869).

### 3.4 Microstate Mean Occurrence

At the Bonferroni‐adjusted significance level of 0.007, no comparisons of mean occurrences for each of the seven microstates between pre‐ and post‐chemotherapy were statistically different. Based on the observed large effect size, however, results of practical significance are the reduction in the mean occurrence of microstate B (*p = *0.018*, d = *1.090) and microstate F (*p = *0.029*, d = *0.971). Moreover, the mean occurrence rate of microstates E (*p = *0.177*, d = *0.531) and C decreased (*p = *0.256*, d = *0.438) with medium and small effect sizes, respectively. The mean occurrence rate of microstate D increased following chemotherapy, but the effect size was negligible and was not statistically significant (*p = *0.765*, d = *0.110).

### 3.5 Correlation Analysis

Among the microstate features studied, six microstate features displayed a large effect size. Among the measures of cognitive function studied, the PROMIS Applied Cognitive Abilities Scales had a large effect size, while this was not the case for the other measures of the objective cognitive outcomes (Table 2). Thus, six selected microstate features (i.e., the mean durations of microstates A, C, D, and G, and the mean occurrences of microstates B and F) were correlated with the PROMIS score at the pre‐ and post‐chemotherapy timepoints. Using a Bonferroni‐adjusted significance level of 0.008 to account for the increased probability of type‐I error, none of the estimated correlations approached statistical significance at either timepoint. Given the maximum observed Pearson correlation coefficient of 0.351, the small sample size, and the potential for random variation, the relationships were considered too weak to report and were not deemed to contribute meaningfully to the interpretation of the primary results. Moreover, there were negligible differences in the magnitudes of the correlation coefficients when comparing pre‐ and post‐chemotherapy timepoints.

## 4 Discussion

In this exploratory study, we investigated changes in RS EEG microstates and explored potential associations with cognitive function in a within‐sample cohort of eight individuals with breast cancer. Despite the small sample size, significant changes were observed in both EEG microstates and self‐perceived cognitive function. As expected based on prior research (Anderson‐Hanley et al. 2003; Henderson, Cross, and Baraniak 2019), our participants self‐reported significantly greater cognitive impairments following chemotherapy for breast cancer. No significant changes were observed in TMT performance, which instead showed slight improvement. Given participants’ self‐reported cognitive impairment, it is unlikely these improvements represent cognitive gains. The discrepancy may be explained by altered strategies, increased effort, increased familiarity with TMT at the post‐chemotherapy timepoint, or cognitive reserve (i.e., the brain's potential to actively compensate for cognitive impairment). The changes we observed in the microstates post‐chemotherapy may shed light on possible explanations between self‐reported cognitive impairment and objective cognitive performance.

Our most significant finding was the increase in the mean duration of microstate D following chemotherapy. Microstate D is associated with attention, orientation, and executive function RS networks (e.g., right inferior parietal lobe and right middle and superior frontal gyri) (Custo et al. 2017; Britz, Van De Ville, and Michel 2010). Patients with breast cancer frequently report impaired attention and executive function following chemotherapy (Kam, Brenner, et al. 2016; Henneghan and Kesler 2023). A disruption in these cognitive functions is associated with symptoms of mental fatigue, difficulties with attention, and memory impairment (Arya et al. 2021). The increased duration of microstate D activation may reflect compensatory hyperactivity in response to increased cognitive demands, particularly in attention and executive function (Arya et al. 2021). The increased effort may have masked declines in objective performance.

These results build upon prior fMRI research (Kam, Boyd, et al. 2016) and the review of fMRI studies conducted by Arya et al. (2021), which links CRCI with frontoparietal hyperactivation (i.e., functional areas related to executive function). Whereas Arya et al. (2021) used cross‐sectional data, our study adds new evidence using a within‐subject design showing heightened activity in these cortical areas post‐chemotherapy relative to baseline.

Our findings with respect to microstates C and F were mixed. While the mean duration of microstate C increased (with a large effect size) post‐chemotherapy, we observed a small decrease in the mean occurrence of microstate C. This aligns with our general observation that all microstate episodes were more prolonged following chemotherapy. Microstate C has been associated with the salience RS network as well as functional areas associated with self‐referential thought (Britz, Van De Ville, and Michel 2010; Tarailis et al. 2023). This finding may add further support to the suggestion that changes in salience network connectivity may be a useful objective biomarker for CRCI in breast cancer survivors (Henneghan and Kesler 2023). In contrast, Microstate F showed a large decrease in mean occurrence but an increase in duration relative to baseline, possibly reflecting disruptions in the DMN following chemotherapy. Microstate F has been associated with DMN activity (Tarailis et al. 2023), and this aligns with the observation that several DMN connections may be disrupted following chemotherapy (Arya et al. 2021; Phillips et al. 2022).

Our preliminary findings in microstates A, B, E, and G suggest potential changes in sensory processing and networks related to cognition. Britz, Van De Ville and Michel (2010) and Custo et al. (2017) have shown microstate A is primarily associated with the auditory processing RSN and language areas (e.g., left middle and superior temporal lobe, including primary auditory cortex and left Wernicke area), and microstate B is primarily associated with the visual imagery RSN (e.g., left and right occipital cortices, including primary visual cortex). A recent review by Tarailis et al. (2023) suggested microstate A may also be related to visual processing and arousal brain areas. Microstate topographies E and G have also been shown to be associated with various functional brain networks. Tarailis et al. (2023) argue that microstate E (more so than microstate C) shows a strong association with the salience network, interoception, and the processing of autonomic information. Microstate G, on the other hand, may primarily be associated with somatosensory network activity (Tarailis et al. 2023).

The increase in the mean duration of microstate A but reduction in the mean occurrence of microstate B following chemotherapy (both results nearly attained significance and exhibited a large effect size) points to reduced activity in visual processing and imagery brain areas but increased activity in auditory and language processing areas following chemotherapy (Custo et al. 2017; Britz, Van De Ville, and Michel 2010). We also observed an increase in the mean duration of microstate G following chemotherapy, a microstate associated with somatosensory network activity (Tarailis et al. 2023). Perhaps this altered balance of sensory processing activity in the brain relates to the cognitive impairments experienced by our breast cancer cohort with chemotherapy. Future research should further explore, as a primary research question, how chemotherapy alters the processing of various sensory inputs in the brain and whether an imbalance in brain activity related to the processing of different sensory inputs is a cause or effect of symptoms such as mental fatigue, attentional deficits, and memory impairment.

In a healthy brain, individuals display a typical pattern of shifting between different brain states at rest, spending roughly equal amounts of time in each state (Muller and Virji‐Babul 2018). Given our finding of longer and less evenly distributed microstate durations post‐chemotherapy, our tentative conclusion is that together, these changes may reflect the neural basis of “brain fog” characterized by difficulties in shifting between cognitive states, such as shifting one's attention from an internal state to paying attention to the external world (Muller and Virji‐Babul 2018). We have previously observed similar changes in a cohort of individuals with concussion who also exhibited and complained of “brain fog” in the first few weeks post‐concussion (Muller and Virji‐Babul 2018; Sattari et al. 2024). Barzon et al. (2024) have recently shown that as cognitive tasks become more demanding, there is an observable shift in EEG microstates; importantly, these shifts are associated with higher metabolic costs with a reorganization of neural dynamics associated with increased cognitive effort. This new perspective has not yet been applied to conditions that are associated with brain fog but provides an intriguing, testable hypothesis that brain fog, regardless of the underlying causes (i.e., chemotherapy or concussion), may stem from a common disruption of the brain network dynamics which results in compensatory network reorganization that behavioral manifests as “brain fog.” Further research with a larger cohort of participants would be invaluable in unravelling the shared neural disruptions related to this phenomenon and potentially guide the development of targeted treatments across different conditions.

The main limitations of this exploratory study are the small sample size, the lack of a control group for comparison due to collapsing the study arms into one group for the purposes of analysis, and the number of statistical tests performed, leading us to employ a conservative multiple testing correction, which made statistical significance a less relevant outcome measure than measures of practical significance (i.e., standardized effect size). For our analysis, all participants were collapsed into a single group as the goal of this pilot investigation was to observe differences before and after chemotherapy. It is possible that changes could be due to task familiarity with the TMT and PROMIS and the potential effect of undertaking aerobic exercise during chemotherapy. In addition, we did not differentiate between patients who received ACT (doxorubicin, cyclophosphamide, paclitaxel), ACT‐G (agents in ACT + filgrastim), and DC (docetaxel, cyclophosphamide), which may have differential effects on brain responses (Vasaghi Gharamaleki et al. 2022). Other forms of cancer treatment, such as immunotherapy and radiotherapy, may also give rise to cognitive deficits that warrant further EEG microstate investigation (Schagen et al. 2022).

## 5 Conclusions

This exploratory study provides evidence for disturbed functional dynamics in the RS brain following chemotherapy and introduces EEG microstates as a potential biomarker to evaluate the changes in brain dynamics related to the cognitive effects of chemotherapy. Further research is needed to confirm these novel exploratory findings.

## Author Contributions

SD and SS conducted the analysis of the EEG data and the statistical analysis, drafted sections of the manuscript and critically revised and edited the manuscript. KZ assisted with the analysis of the behavioural data and critically reviewed and edited the manuscript. KLC and JB conceptualized the ACTIVATE trial, acquired funding, oversaw the conduct of the trial and critically reviewed and edited the manuscript. NVB contributed to the EEG trial design and critically reviewed and edited the manuscript. All authors read and approved the final manuscript.

## Ethics Statement

Ethics approval for the ACTIVATE trial was granted by the research ethics boards at the University of Ottawa (Ottawa, ON) and the University of British Columbia (UBC) (Vancouver, BC), as well as relevant hospital research ethics committees. The REB number for the data collected in Vancouver is H17‐00563 (UBC/BC Cancer research ethics boards). All participants received written and oral information prior to participation and provide informed consent.

### Peer Review

The peer review history for this article is available at https://publons.com/publon/10.1002/brb3.70335.

## Data Availability

The raw data supporting the conclusions of this manuscript cannot be made available by the authors as patients were assured their data would be kept private and confidential to the extent permitted by law and that only the research team would have access to the data.
